# First-line serplulimab plus chemotherapy versus chemotherapy in PD-L1-positive esophageal squamous-cell carcinoma: a cost-effectiveness analysis

**DOI:** 10.1038/s41598-024-65474-7

**Published:** 2024-06-24

**Authors:** Xueyan Liang, Mingyu Meng, Shiran Qin, Xiaoyu Chen, Yan Li

**Affiliations:** 1 Phase 1 Clinical Trial Laboratory, Guangxi Academy of Medical Sciences and the People’s Hospital of Guangxi Zhuang Autonomous Region, Nanning, Guangxi People’s Republic of China; 2 Department of Pharmacy, Guangxi Academy of Medical Sciences and the People’s Hospital of Guangxi Zhuang Autonomous Region, Nanning, Guangxi People’s Republic of China

**Keywords:** Serplulimab, Esophageal squamous cell carcinoma, Cost-effectiveness, Chemotherapy, Partitioned survival model, Cancer immunotherapy, Chemotherapy

## Abstract

Patients with PD-L1-positive esophageal squamous-cell carcinoma (ESCC) were significantly more likely to survive when treated with serplulimab plus cisplatin plus 5-fluorouracil (serplulimab-CF). At this point, it is unknown whether this expensive therapy is cost-effective. From the Chinese healthcare system's perspective, we aimed to evaluate serplulimab-CF versus CF alone for cost-effectiveness. A partitioned survival model was constructed based on the ASTRUM-007 trial. Quality-adjusted life-years (QALYs) and incremental cost-effectiveness ratio (ICER) were calculated. A further analysis of subgroups and scenarios was conducted. The willingness to pay (WTP) threshold of $38,258/QALY or $84,866/QALY is defined as three times the per capita gross domestic product value of the general region or affluent region. Compared with CF alone, in the overall (scenario 1), patients with PD-L1 expression level of 1 ≤ CPS < 10 (scenario 2), and patients with PD-L1 CPS ≥ 10 (scenario 3) populations, the ICERs were $69,025/QALY, $82,533/QALY, and $75,436/QALY for serplulimab-CF. Nevertheless, the probability of serplulimab-CF becoming cost-effective based on scenarios 1, 2, and 3 is only 2.71%, 0.94%, and 2.84%, respectively, at a WTP threshold of $38,258/QALY. When serplulimab costs < $4.84/mg, serplulimab-CF may be cost-effective at the WTP threshold of $38,258/QALY; otherwise, CF was preferred. Similar results were obtained from sensitivity analyses, suggesting the robustness of these findings. There was no cost-effectiveness in general regions of China for serplulimab-CF in PD-L1-positive ESCC compared to CF, although it is probably considered cost-effective in affluent regions. Serplulimab-CF may achieve favorable cost-effectiveness by lowering the price of serplulimab.

## Introduction

The incidence and mortality of esophageal cancer are ranked seventh and sixth globally, respectively, and most cases occur in East Asian countries^1^. Nearly 84% of all cases of esophageal cancer are caused by esophageal squamous-cell carcinoma (ESCC), which is the most frequent histological subtype^2^. For patients with advanced, recurrent or metastatic ESCC, standard platinum plus fluorouracil or paclitaxel-based chemotherapy are recommended as first-line treatments. The median overall survival (OS) among patients with advanced ESCC remains poor despite advances in chemotherapy, typically 10–12 months^3,4^. A novel treatment strategy is therefore needed.

Treatment options for ESCC have improved significantly since immunotherapeutic treatment regimens were introduced. As a result of advances in immunotherapy, programmed cell death protein ligand 1 (PD-L1) has been identified as a potential target and biomarker for patients with ESCC. It has been reported that up to 40% of ESCCs have overexpressed PD-L1^5^. Researchers have also found a positive association between PD-L1 expression and outcomes with PD-1 inhibitors plus chemotherapy in phase 3 randomized trials of advanced ESCC^3,4^. Patients with ESCC who were not selected for PD-L1 expression appeared to benefit from PD-1 inhibitors in three clinical trials in phase 3, with objective response rates (ORRs) ranging from 16.7 to 20.2%^6–8^. This is a significant improvement and represents a major improvement in the treatment of this disease, but further innovation is needed to produce satisfactory OS outcomes because improved long-term survival is crucial for allowing patients more salvage treatments to be performed.

A monoclonal antibody against the PD-1 receptor, serplulimab is a fully humanized and selective immunoglobulin G4 antibody. In China, Serplulimab was approved for the first time on 25 Mar 2022 to treat unresectable or metastatic solid tumours with microsatellite instability-high (MSI-H) that are unresponsive to previous standard treatments^9^. Recently, the results of ASTRUM-007 trial, which assessed the efficacy and safety of serplulimab plus cisplatin plus 5-fluorouracil (serplulimab plus CF) versus cisplatin plus 5-fluorouracil (CF) alone as a first-line treatment in patients with advanced or metastatic ESCC with PD-L1 combined positive score (CPS) ≥ 1^10^. According to the trial, serplulimab plus CF resulted in an OS improvement (hazard ratio [HR] 0.68; 95% confidence interval [CI] 0.53–0.87) compared to CF alone, along with an improvement in progression-free survival (PFS, HR 0.60; 95% CI 0.48–0.75). ASTRUM-007 demonstrated significant improvements in PFS and OS for patients with previously untreated, PD-L1-positive advanced ESCC when prescribed serplulimab plus CF treatment. The safety profile was manageable.

Innovative drugs are always associated with significant costs. Availability of serplulimab may be limited by its high cost, imposing a significant financial burden on the national health system. Although serplulimab has greatly improved the survival of ESCC patients when combined with CF, further research is needed to determine whether this combination is economically viable and what populations will benefit from it. As a result, this study assessed the cost-effectiveness of serplulimab plus CF as a first-line treatment option for PD-L1-positive ESCC in China. It may be possible to optimize the utilization of resources by incorporating such evidence into clinical practices and reimbursement policies.

## Methods

### Patients and intervention

The ASTRUM-007 trial^10^ was the basis for this economic evaluation study, and no real human participants involved in this study, so no institutional review board approval was required. Consolidated Health Economic Evaluation Reporting Standards (CHEERS)  2022 were followed for the reporting in this study^11^. As in the ASTRUM-007 trial, the target patients remained the same. The target patients were kept with the cohort included in the ASTRUM-007 trial. Patients with PD-L1-positive (CPS ≥ 1), previously untreated, inoperable locally advanced or metastatic ESCC, and whose aged at least 18 years are eligible for this trial.

Serplulimab plus chemotherapy or placebo plus chemotherapy was administered randomly to the included patients. Serplulimab or placebo was given at a dose of 3 mg/kg once every two weeks for as long as two years to patients. All patients received intravenous doses of cisplatin (50 mg/m^2^) and 5-FU (1,200 mg/m^2^) on days 1 and 2 of every two weeks. The patient was treated until disease progression, intolerable toxicities, or death. A total of 139 (38%) patients in the serplulimab plus CF group and 95 (52%) in the CF group received subsequent anticancer therapy; 64 (17%) in the serplulimab plus CF group and 61 (33%) in the CF group received subsequent immunotherapy in the ASTRUM-007 trial. Considering the limited details provided in the preliminary trial, we adopted subsequent treatment strategies recommended by the National Comprehensive Cancer Network (NCCN)^12^ and the Chinese Society of Clinical Oncology (CSCO) guidelines^13^.

### Partitioned survival model

The two competing regimens for patients with ESCC were compared using a partitioned survival model (PSM). Three health states were considered in the model: progressed disease (PD), progression-free survival (PFS), and death. Each patient was assigned a PFS state, and they were able to maintain their assigned states or re-distribute to another. Area under the curve (AUC) for PFS was used for estimating the proportion of patients in the PFS state, while 1 minus the OS curve was used to estimate the proportion of patients in the death state. PD states are determined by the AUC between OS and PFS curves. Parameter calculations were simplified by setting the cycle length to one week. The time horizon intended to achieve terminal status for ESCC was 15 years (more than 99% of patients die within this timeframe).

A Chinese healthcare system perspective was taken in this study. The primary outcomes included life-years, cost, quality-adjusted life-years (QALYs), along with incremental net health benefit (INHB) and incremental net monetary benefit (INMB). ICER is the additional cost incurred for each additional QALY. A 5% discount rate per year was used for both costs and QALYs^14^. The cost of each item was adjusted according to the local Consumer Price Index and converted into US dollars (1$ = 6.72 CNY). According to World Health Organization (WHO) recommendations, we determined cost-effectiveness by using 3 times China's per capita gross domestic product (GDP) in 2022 as a willingness to pay threshold (WTP)^15,16^. The ICERs have been calculated with two WTP thresholds to account for the uneven development of economic activity in China's various socioeconomic regions: three times the per capita GDP value of China in 2022 (USD 38,258/QALY) for general regions and three times the per capita GDP value of Beijing city in 2022 (USD 84,866/QALY) for affluent regions^17^. ICERs less than one time GDP per capita were considered highly cost-effective, while those less than three times GDP per capita were considered cost-effective^15^. There has been widespread use of this WTP threshold in low- and middle-income countries when assessing health technology^18^.

### Clinical data inputs

Efficacy and safety data were derived from the ASTRUM-007 trial^10^. We extracted PFS and OS data points from Kaplan–Meier (K–M) survival curves using GetData Graph Digitizer version 2.26^19^ because individual patient data (IPD) were not available. An extrapolation of the survival curves beyond the follow-up duration of clinical trials was achieved using a variety of parametric distributions, such as an Exponential distribution, a Weibull distribution, a Gamma distribution, a Lognormal distribution, a Gompertz distribution, a Log-logistic distribution, and a Generalized Gamma distribution. On the basis of the Akaike and Bayesian information criteria (AIC and BIC), the best fit distribution was selected (Supplementary Table 1). In Supplementary Fig. 1, we show the model-fitted versus original K-M curves for serplulimab and CF or CF. R version 4.0.5 was used for survival analyses to calculate the AIC and BIC. A summary of the key clinical input data can be found in Table 1^10,14,20–31^.

**Table 1:** Key model inputs.

Parameter	Values	Distribution	Source
Scenario 1 overall PD-L1-positive ESCC Patients			
Log-logistic OS survival model of serplulimab plus CF^a^	γ = 1.7290, λ = 0.0148	ND	10
Lognormal PFS survival model of serplulimab plus CF^a^	μ = 3.3373, σ = 0.9854	ND	10
Log-logistic OS survival model of CF^a^	μ = 1.9429, σ = 0.0200	ND	10
Log-logistic PFS survival model of CF^a^	γ = 2.3436, λ = 0.0493	ND	10
Scenario 2 patients with PD-L1 expression level of 1 ≤ CPS < 10			
Log-logistic OS survival model of serplulimab plus CF^a^	γ = 1.6812, λ = 0.0166	ND	10
Log-logistic PFS survival model of serplulimab plus CF^a^	γ = 1.8984, λ = 0.0410	ND	10
Log-logistic OS survival model of CF^a^	γ = 2.1860, λ = 0.0206	ND	10
Log-logistic PFS survival model of CF^a^	γ = 2.4457, λ = 0.4915	ND	10
Scenario 3 Patients with PD-L1 CPS ≥ 10			
Lognormal OS survival model of serplulimab plus CF^a^	μ = 4.3799, σ = 0.9887	ND	10
Lognormal PFS survival model of serplulimab plus CF^a^	μ = 3.5080, σ = 1.0168	ND	10
Log-logistic OS survival model of CF^a^	γ = 1.6515, λ = 0.0186	ND	10
Log-logistic PFS survival model of CF^a^	γ = 2.1759, λ = 0.0492	ND	10
Cost input			
Drug costs per 1 mg			
Serplulimab	8.32 (Range: 6.65–9.98)	Gamma (76, 0.11)	Local database
Cisplatin	0.27 (Range: 0.2–0.34)	Gamma (25, 0.01)	Local database
5-fluorouracil	0.06 (Range: 0.05–0.08)	Gamma (11, 0.006)	Local database
Second-line treatment in serplulimab plus chemotherapy arm per cycle	283 (Range: 227–340)	Gamma (25, 11.32)	10, Local database
Second-line treatment in chemotherapy arm per cycle	474 (Range: 379–569)	Gamma (25, 18.96)	10, Local database
Routine follow-up cost per cycle	18.39 (Range: 13.79–22.99)	Gamma (25, 0.74)	14
Cost of laboratory tests and radiological examinations	357 (Range: 285–428)	Gamma (25, 14.28)	14
Best supportive care per cycle	41.82 (Range: 31.37–52.28)	Gamma (25, 1.67)	14
Cost of terminal care per patient^b^	1460 (Range: 1168–1752)	Gamma (25, 58.40)	20
Cost of managing AEs (grade ≥ 3)^c^			
Sugemalimab plus CF	2201 (Range: 1761–2641)	Gamma (25, 88.04)	21–23
CF	1862 (Range: 1490–2235)	Gamma (25, 74.48)	21–23
Cost of drug administration per unit	20.64 (Range: 15.48–25.8)	Gamma (25, 0.83)	24
Health utilities			
Disease status utility per year			
PFS	0.741 (Range: 0.593–0.889)	Beta (5.73, 2.00)	25, 26
PD	0.581 (Range: 0.465–0.697)	Beta (9.89, 7.14)	25, 26
Death	0	NA	
Disutility due to AEs (grade ≥ 3)^d^			
Sugemalimab plus CF	0.09 (Range: 0.072–0.108)	Beta (22.66, 229)	27–29
CF	0.081 (Range: 0.065–0.098)	Beta (22.89, 260)	27–29
Other parments			
Body surface area, m^2^	1.80 (Range: 1.44–2.16)	Normal (1.80, 0.23)	30, 31
Body weight, kg	65 (Range: 50–90)	Normal (65, 10)	30, 31
Creatinine clearance rate, ml/min/1.73m^2^	90 (Range: 80–100)	Normal (90, 10)	30

*AE* Adverse event, *CF* Cisplatin plus 5-fluorouracil, *CPS* Combined positive score, *OS* Overall survival, *PD* Progressed disease, *PD-L1* Programmed cell death protein ligand 1, *PFS* Progression-free survival.

^a^Only expected values are presented for these survival model parameters.

^b^Overall total cost per patient regardless of treatment duration.

^c^Calculated as the average cost of toxic effects using weighted frequencies of grade ≥ 3 treatment related adverse events for each treatment arm in the ASTRUM-007 trial. Costs of individual toxic effects were derived from the literature and include all care required to manage each toxic effect. References for individual toxic effect costs are summarized in Supplementary Table 2.

^d^Calculated as the average disutility of toxic effects using weighted frequencies of grade ≥ 3 treatment-related adverse events for each treatment arm in the ASTRUM-007 trial. Disutilities of individual toxic effects were derived from the literature. References for individual toxic effect disutilities are summarized in Supplementary Table 2.

### Costs

During this analysis only direct medical costs such as medication costs, laboratory tests, and radiological examinations were considered, as well as the cost of routine follow-up, the management of adverse events (AEs) associated with treatment, and best supportive care during the end stages of the illness. The drug costs were obtained from the local database of the Guangxi Academy of Medical Sciences and the People’s Hospital of Guangxi Zhuang Autonomous Region. Accordingly, we assumed 90 ml/min/1.73 m^2^, 65 kg, and 1.80 m^2^, respectively, for the creatinine clearance rate (Ccr), body weight, and average body surface area (BSA), to estimate therapeutic drug dosage^30,31^. Grade ≥ 3 AEs were included in the model when they occurred in more than 3% of patients, as they have a significant impact on survival and cost. A list of all costs was compiled from local hospitals or previous publications.

### Utilities

In this PSM, each state of health was assigned a utility value between 0 (death) and 1 (perfect health). We derived utility values from the published literature since the ASTRUM-007 trial did not report utility values for different health states. With regard to ESCC, the health utilities of PD and PFS were respectively 0.581 and 0.741^25,26^. A disutility value was also calculated for grade ≥ 3 AEs based on the available literature (Supplementary Table 2)^21–23,27–29^.

### Sensitivity analysis

A one-way sensitivity analysis was performed for each input parameter to evaluate the robustness of the model. Using a range of the 95% confidence intervals (CIs) reported in the referenced literature or a change of 20% from the base-case value, the input parameters were adjusted one by one to their minimum and maximum values, so that we could determine which variables significantly influenced economic outcomes.

In order to perform a probabilistic sensitivity analysis, a Monte Carlo simulation was run with 10,000 iterations, sampling all parameters at the same time from prespecified distributions. Gamma distribution was used to sample all the costs. Beta distribution was used to sample utility values and probabilities. To illustrate the probability of cost-effectiveness of serplulimab plus CF compared with CF alone at different WTP thresholds, cost-effectiveness acceptability curves (CEAC) were plotted based on the results of 10,000 iterations.

### Subgroup analysis

An analysis of subgroups was also conducted to investigate the effect of different characteristics on outcomes. By varying the HRs for OS and PFS, subgroup analyses were performed for the different subgroups derived from the ASTRUM-007 study. R packages hesim and heemod, versions 4.0.5, were used to perform statistical analyses.

### Ethical approval and informed consent statement

This is a study based on secondary data without primary patient data collection, and therefore, no ethical approval is required. According to the Guangxi Academy of Medical Sciences and the People’s Hospital of Guangxi Zhuang Autonomous Region, since publicly available data from the literature and local database of the Guangxi Academy of Medical Sciences and the People’s Hospital of Guangxi Zhuang Autonomous Region were used to conduct this study rather than individual patient level data, institutional review board review and informed consent were not required nor obtained.

## Results

### Base-case results

#### Scenario 1 Regarding overall patients with PD-L1-positive advanced ESCC

Serplulimab-CF provided an incremental cost of $28,585 with an additional 0.414 QALYs, resulting in an ICER of $69,025/QALY. At a $38,258/QALY WTP threshold compared with CF alone, the INHB and INMB of serplulimab-CF were -0.333 QALYs and -$12,742, respectively. Moreover, at a $84,866/QALY WTP threshold, the INHB and INMB of serplulimab-CF were 0.077 QALYs and $6,560, respectively (Table 2).

**Table 2:** Summary of cost and outcome results in the base-case analysis.

Factor	Scenario 1 Overall PD-L1-positive ESCC patients			Scenario 2 Patients with PD-L1 expression level of 1 ≤ CPS < 10			Scenario 3 Patients with PD-L1 CPS ≥ 10		
	Serplulimab plus CF	CF	Incremental change	Serplulimab plus CF	CF	Incremental change	Serplulimab plus CF	CF	Incremental change
*Cost, $*									
Drug^a^	48,797	22,924	25,873	43,633	20,384	23,249	53,682	27,729	25,953
Nondrug^b^	9,440	6,728	2,712	8,244	6,373	1,871	10,079	7,429	2,650
Overall	58,237	29,652	28,585	51,877	26,757	25,120	63,761	35,158	28,603
*Life-years*									
Progression-free	0.980	0.532	0.448	0.744	0.520	0.224	1.059	0.561	0.498
Overall	2.182	1.440	0.742	1.871	1.301	0.570	2.332	1.713	0.619
QALYs	1.269	0.855	0.414	1.092	0.787	0.305	1.366	0.987	0.379
*ICERs, $*									
Per life-year	NA	NA	38,536	NA	NA	40,809	NA	NA	46,253
Per QALY	NA	NA	69,025	NA	NA	82,533	NA	NA	75,436
INHB, QALY, at threshold 38,258^a^	NA	NA	− 0.333	NA	NA	-0.352	NA	NA	-0.368
INMB, $, at threshold 38,258^a^	NA	NA	-12,742	NA	NA	-13,476	NA	NA	-14,097
INHB, QALY, at threshold 84,866^a^	NA	NA	0.077	NA	NA	0.008	NA	NA	0.042
INMB, $, at threshold 84,866^a^	NA	NA	6,560	NA	NA	710	NA	NA	3,576

*CF* Cisplatin plus 5-fluorouracil, *CPS* Combined positive score, *ICER* Incremental cost-effectiveness ratio, *INHB* Incremental net health benefit, *INMB* Incremental net monetary benefit, *NA* Not applicable, *PD-L1* Programmed cell death protein ligand 1, *QALYs* Quality-adjusted life-years.

^a^Compared with CF.

^b^Nondrug cost includes the costs of adverse event management, subsequent best supportive care per patient, and follow-up care covering physician monitors and terminal care.

#### *Scenario 2 Regarding patients with PD-L1 expression levels of 1* ≤ *CPS* < *10*

In comparison with CF alone, serplulimab-CF provided an additional 0.305 QALYs and 0.570 overall life-years, with an incremental cost of $25,120, which was associated with an ICER of $82,533/QALY. The INHB was -0.352 QALYs, and the INMB was -$13,476 at a WTP threshold of $38,258/QALY (Table 2).

#### *Scenario 3 Regarding patients with PD-L1 CPS* ≥ *10*

Serplulimab-CF resulted in an increase of 0.379 QALYs effectiveness and 0.619 overall life-years vs. CF alone, as well as an additional cost of $28,603. The corresponding ICER was $75,436/QALY. Moreover, at a $38,258/QALY WTP threshold, the INHB and INMB of serplulimab-CF were -0.368 QALYs and -$14,097, respectively. At a WTP threshold of $84,866/QALY, the INHB and INMB were 0.042 QALYs and $3,576, respectively (Table 2).

In all three scenarios, the ICERs were lower than the WTP threshold of $84,866/QALY (for affluent regions in China) but showed considerably higher than the $38,258/QALY WTP threshold (for general regions in China). The results showed that serplulimab-CF was unlikely to be a cost-effective first-line treatment option for patients with PD-L1-positive advanced ESCC in general regions of China, when compared with CF alone. It was evaluated in affluent regions of China, however, that serplulimab-CF would be cost-effective.

### Sensitivity analysis

According to the one-way sensitivity analysis, body weight, price of serplulimab, and HR of OS for the serplulimab-CF arm affected the outcomes most sensitively. There was only a moderate or marginal association between the remaining parameters and the model (Supplementary Fig. 2).

**Figure 1:**
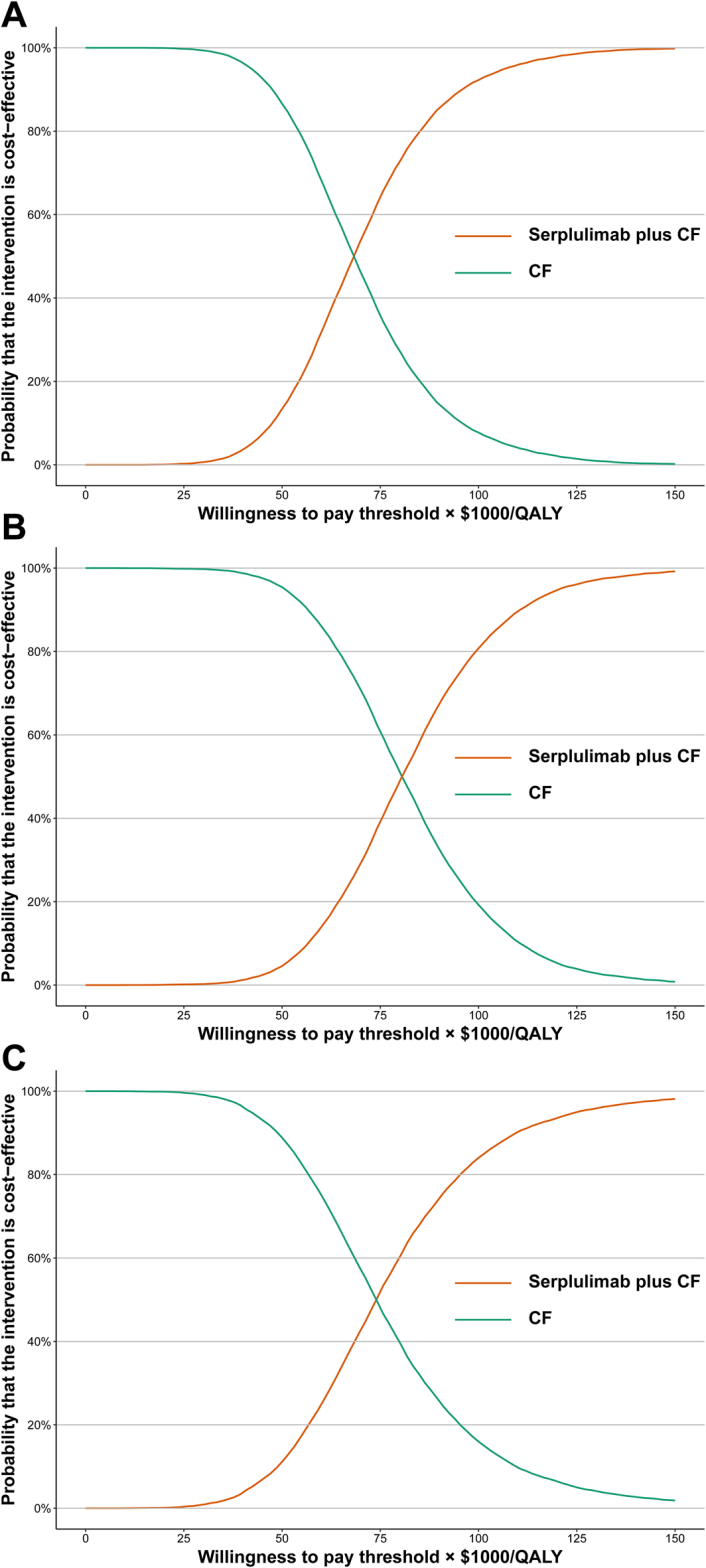
Acceptability curves of cost-effectiveness for serplulimab plus CF versus CF. (**A**) overall PD-L1-positive ESCC patients, (**B**) patients with PD-L1 expression level of 1 ≤ CPS  < 10, (**C**) patients with PD-L1 CPS ≥ 10. CF, cisplatin plus 5-fluorouracil; CPS, combined positive score; PD-L1, programmed cell death protein ligand 1; QALYs, quality-adjusted life-years.

In scenarios 1, 2, and 3, the probability of serplulimab-CF treatment being cost-effective was 79.69%, 58.09%, and 67.83%, respectively, for the WTP threshold of 84,866/QALY and current price. In spite of this, serplulimab-CF had less than 3% probability of being considered cost-effective at the WTP threshold of $38,258/QALY for scenarios 1 (2.71%), 2 (0.94%), and 3 (2.84%) (Fig. 1).

The relationship between these key variables and the ICER was also examined. At a WTP threshold of 84,866 QALYs, serplulimab-CF was cost-effective for patients with an average body weight < 78.96 kg or a price of serplulimab < $10.10/mg. This result was consistent with base-case results under current price of serplulimab ($8.32/mg, 95% CI 6.65–9.98), of which serplulimab-CF be cost-effective compared with CF alone for affluent regions (a WTP threshold of $84,866/QALY). Crucially, serplulimab-CF may be cost-effective at the WTP threshold of $38,258/QALY when a price of serplulimab < $4.84/mg; otherwise, CF was preferred (Supplementary Fig. 3).

### Subgroup analysis

By varying the HRs for OS and PFS, a subgroup analysis was performed. It was found that serplulimab-CF reduced better at the risk of death for most subgroups. Generally, subgroups with better survival advantages were found to have a higher likelihood of being cost-effective. ICERs were lower than the WTP threshold of $84,866/QALY in all subgroups, but considerably higher than the threshold of $38,258/QALY. Furthermore, the probability of serplulimab-CF being considered cost-effective was > 50% in all subgroups reported and > 80% in most subgroups at a WTP threshold of $84,866/QALY. However, in all subgroups evaluated, the probabilities being considered cost-effective were < 20% at a WTP threshold of $38,258/QALY. Serplulimab-CF was associated with positive INHBs in all subgroups at the WTP threshold of $84,866/QALY, nevertheless, the values of INHBs were negative in all subgroups at the WTP threshold of $38,258/QALY. These results were consistent with base-case results (Table 3).

**Table 3:** Summary of subgroup analyses obtained by varying the hazard ratios (HRs) for PFS and OS.

Subgroup	Unstratified hazard ratio (95% CI)	Change in cost, $^a^	Change in QALYs^a^	ICER, $/QALY	Cost-effectiveness probability of serplulimab plus CF, %		INHB, QALY	
					WTP of $38,258/QALY	WTP of $84,866/QALY	WTP of $38,258/QALY	WTP of $84,866/QALY
PFS subgroup								
Age (years)								
< 65	0.61 (0.45–0.83)	28,455	0.411	69,172	2.69	79.26	− 0.332	0.076
≥ 65	0.57 (0.41–0.81)	28,968	0.423	68,453	3.23	79.95	− 0.334	0.082
ECOG performance status								
0	0.49 (0.30–0.81)	29,839	0.454	65,750	6.44	80.37	− 0.326	0.102
1	0.65 (0.50–0.85)	27,918	0.401	69,567	2.21	80.35	− 0.328	0.072
Sex								
Male	0.59 (0.46–0.75)	28,715	0.417	68,857	2.93	80.20	− 0.334	0.079
Female	0.41 (0.19–0.85)	30,279	0.498	60,823	13.78	81.42	− 0.294	0.141
Disease status								
Locally advanced	0.71 (0.35–1.44)	27,099	0.389	69,685	1.92	79.76	− 0.319	0.070
Distantly metastatic	0.58 (0.46–0.74)	28,843	0.420	68,667	3.41	80.19	− 0.334	0.080
Smoking status								
Current or former smoker	0.55 (0.42–0.74)	29,210	0.430	67,950	3.99	79.74	− 0.334	0.086
Never smoked	0.64 (0.43–0.97)	28,053	0.404	69,495	2.42	79.97	− 0.330	0.073
OS subgroup								
Age (years)								
< 65	0.62 (0.45–0.87)	31,760	0.518	61,310	4.24	91.65	− 0.312	0.144
≥ 65	0.76 (0.52–1.12)	25,049	0.298	83,947	1.80	53.66	− 0.356	0.003
ECOG performance status								
0	0.43 (0.24–0.78)	46,050	0.986	46,720	17.87	99.60	− 0.218	0.443
1	0.70 (0.53–0.93)	27,633	0.383	72,157	2.39	73.87	− 0.339	0.057
Sex								
Male	0.67 (0.51–0.88)	29,081	0.430	67,577	3.16	82.63	− 0.330	0.088
Female	0.47 (0.21–1.03)	42,374	0.865	48,967	13.71	99.38	− 0.242	0.366
Disease status								
Locally advanced	0.52 (0.26–1.04)	38,334	0.733	52,287	9.09	98.46	− 0.269	0.281
Distantly metastatic	0.70 (0.54–0.92)	27,633	0.383	72,157	2.36	74.03	− 0.339	0.057
Smoking status								
Current or former smoker	0.65 (0.47–0.89)	30,110	0.464	64,889	3.16	86.67	− 0.323	0.109
Never smoked	0.73 (0.47–1.14)	26,292	0.339	77,540	2.26	64.61	− 0.348	0.029

*CF* Cisplatin plus 5-fluorouracil, *ECOG* Eastern Cooperative Oncology Group, *HR* Hazard ratio, *ICER* Incremental cost-effectiveness ratio, *OS* Overall survival, *PFS* Progression-free survival, *QALY* Quality-adjusted life-year, *WTP* Willingness to pay.

^a^Change in cost and change in QALYs represent the results of serplulimab plus CF minus CF.

### Relevant guidelines and regulations statement

Consolidated Health Economic Evaluation Reporting Standards (CHEERS) 2022 were followed for the reporting in this study.

## Discussion

As far as we know, this is the first study to incorporate the latest evidence from a Chinese healthcare system perspective into a modeling analysis of serplulimab combination therapy in the treatment of advanced ESCC. Based on the latest GDP estimates, serplulimab-CF therapy can provide higher health outcomes with higher health expenditures. The ICER for the treatment is well above the WTP threshold of $38,258/QALY. In affluent regions of China (such as Beijing or Shanghai), serplulimab-CF was estimated to be a cost-effective first-line treatment for PD-L1-positive advanced ESCC. The robustness of the model results was confirmed by sensitivity analyses.

China is one of the world's most populous countries due to its large population and rapid economic development. It is therefore objectively true that provincial economies are not developing equally. Taking regional economic disequilibrium into account, the WTP thresholds were set at $38,258/QALY in general regions and $84,866/QALY in affluent regions. Due to higher ICERs than the threshold WTP of $38,258 per QALY, serplulimab-CF may not provide a cost-effective alternative to CF alone. In the affluent regions of China, serplulimab-CF, however, could be considered a cost-effective treatment option. Serplulimab's price and average body weight were both found to affect the model's sensitivity. If the price of serplulimab is less than $4.84/mg, Serplulimab-CF is cost-effective at a WTP threshold of $38,258/QALY; otherwise, CF is preferred.

It is challenging to approve new drugs solely based on survival benefits. The approval of different drugs in different regions should take into account different economic factors. A cost-effective strategy for selecting appropriate patients is crucial for the long-term viability of our healthcare systems, so physicians and administrators need to be aware of the importance of developing these strategies. It is often less cost-effective to combine innovative drugs with existing treatment schemes due to the drastic increase in costs and the uncertainty of survival benefits^32^.

According to our understanding, several rounds of negotiations have been underway on the price of cancer drugs since the National Healthcare Security Administration (NHSA) was established in May 2018. The objective is to reduce cancer patients' medical burden through national strategic procurement^33^. Many anticancer drugs have been reduced by 30–70% because of efforts made by the NHSA in China to negotiate drug prices with pharmaceutical companies^33^. There is little likelihood of serplulimab's price rising in these circumstances. It is highly likely that the cost of serplulimab will decrease if negotiations are conducted for the drug. Consequently, serplulimab-CF provides adequate first-line treatment for patients with PD-L1-positive advanced ESCC within a reasonable price range. As a result of the NHSA negotiation, better health services at lower costs will be provided to Chinese patients for a long period of time^34^. Since there are no further breakthroughs in efficacy at this time, the cost-effectiveness and affordability of treatment options depend on substantial price reductions, especially in countries with high cancer burdens and limited healthcare resources^35^. As a result of our sensitivity analyses, we also found that drug price was a primary factor affecting ICER, and that price reductions could improve the cost-effectiveness of serplulimab-CF.

Several advantages should be highlighted in this study. Firstly, to our knowledge, this is the first study using a PSM based on the latest ASTRUM-007 trial to conduct a cost-effectiveness analysis of serplulimab-CF combination therapy in treating PD-L1-positive advanced ESCC. Second, compared to CF alone, serplulimab-CF combination is unlikely to be cost-effective at current prices. It does, however, appear to improve OS and PFS in patients with PD-L1-positive advanced ESCC. Thirdly, Chinese patients were evaluated in the trial, so race had no influence on the results. It was also possible to partition patients based on the K-M curve directly between health states using the PSM without assuming that patients transition between health states. Finally, 10 subgroups assessed in the ASTRUM-007 trial were analyzed in the present study. The economic results of the subgroups may benefit physicians, policymakers, and patients.

In this analysis, some limitations exist. K-M curves were fitted with parameter distributions based on the ASTRUM-007 trial follow-up period, resulting in uncertain model estimates. The sensitivity analysis indicated that this finding is generally robust, suggesting that this limitation is not significant. Second, clinical data derived from the ASTRUM-007 trial are incorporated into the model. This means that any biases in the trial may have affected its cost-effectiveness results. Patients with advanced ESCC included in the ASTRUM-007 study generally had strict characteristics. A clinical trial participant's treatment regimen will also likely be adhered to more closely than the treatment regimen of a patient in real life. Third, despite the availability of published literature for utility and disutility values, some of the data did not come from Chinese populations.

## Conclusion

At a WTP threshold of $38,258/QALY and under current drug pricing, this study indicates serplulimab-CF may not be cost-effective for treating PD-L1-positive advanced ESCC in China. A substantial price reduction for serplulimab could be economically advantageous. Clinical decision-making may be aided by the results of this study.

### Supplementary Information

Supplementary Information.

## Data Availability

Data is provided within the manuscript or supplementary information files. Specific additional data can be provided upon request to the corresponding author/s.
