# Supplementary material for: First-line serplulimab plus chemotherapy versus chemotherapy in PD-L1-positive esophageal squamous-cell carcinoma: a cost-effectiveness analysis
Source: Sci Rep. 2024 Jun 24;14:14496. doi: 10.1038/s41598-024-65474-7 (PMC11196723; doi:10.1038/s41598-024-65474-7)
Supplement: Supplementary file 1 — Supplementary Information. [file 41598_2024_65474_MOESM1_ESM.pdf]

Supplementary Content

Supplementary Figure 1. Model Fitting Analysis

Supplementary Figure 2. Tornado diagram of one-way sensitivity analyses of serplulimab plus CF versus CF

Supplementary Figure 3. Analyses result of ICER when varying body weight and cost of serplulimab in overall PD-L1-positive ESCC patients.

Supplementary Table 1. Estimated parameters and AIC and BIC values from each survival model.

Supplementary Table 2. Associated costs and disutility of grade ≥ 3 treatment-related adverse events.

(A) Model-fitted versus original K-M curves for serplulimab-CF, Log-logistic model was used to fit the OS and Lognormal model was used to fit the PFS K-M for serplulimab-CF in overall PD-L1-positive patients.

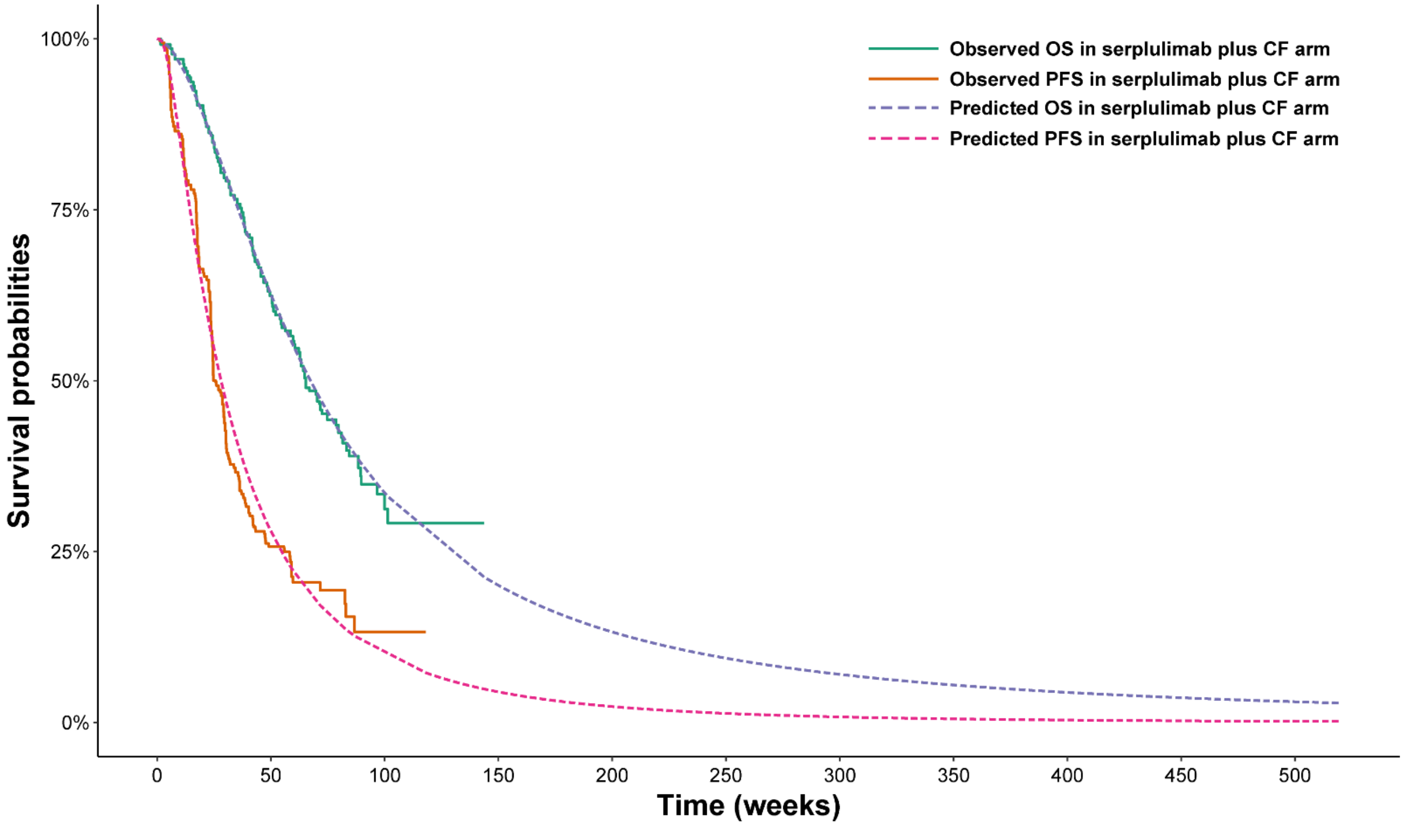

(B) Model-fitted versus original K-M curves for CF, Log-logistic model was used to fit the OS and PFS K-M curves for CF in overall PD-L1-positive patients.

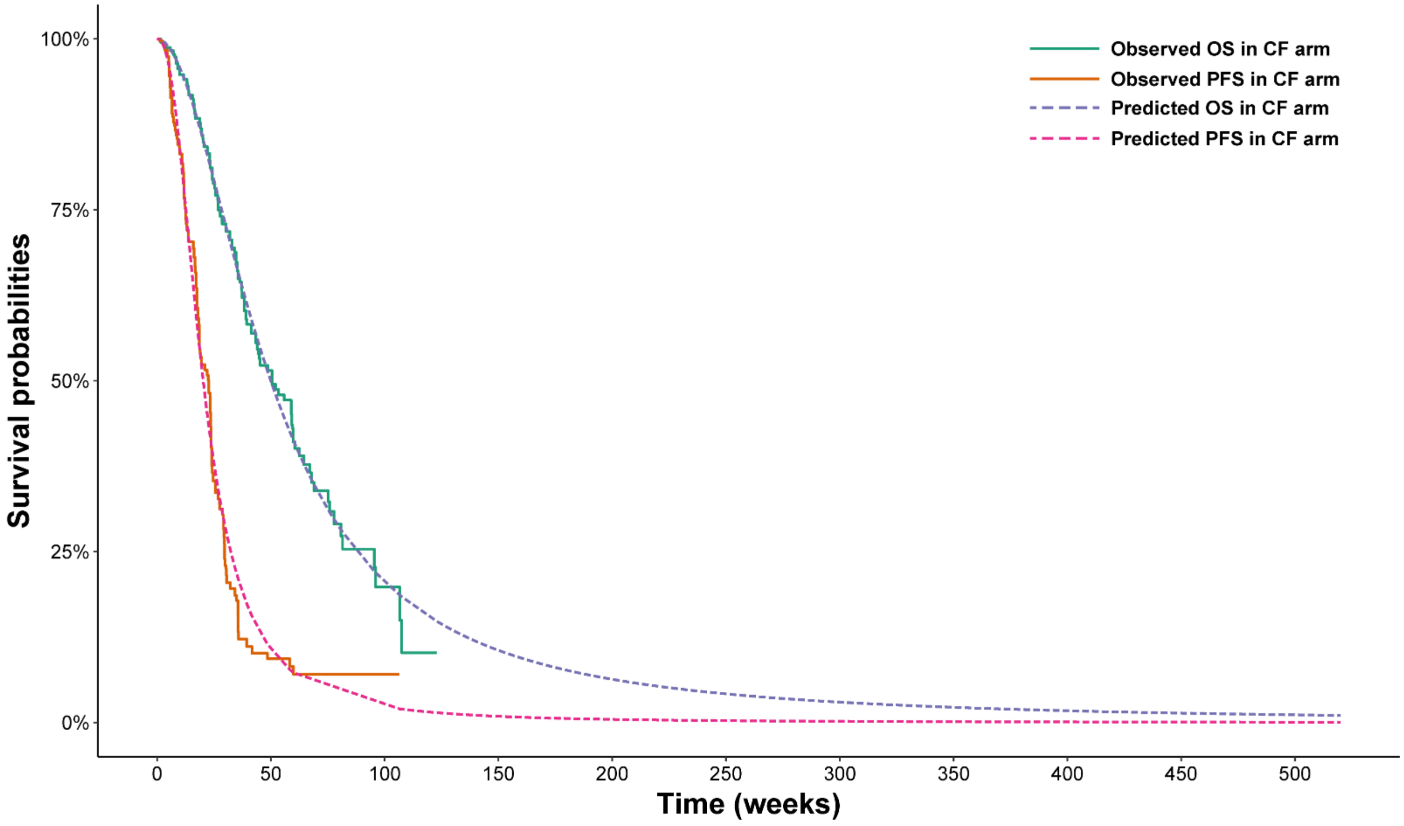

(C) Model-fitted versus original K-M curves for serplulimab plus CF, Log-logistic model was used to fit the OS and PFS K-M curves for serplulimab plus CF in patients with PD-L1 expression level of 1 ≤ CPS < 10.

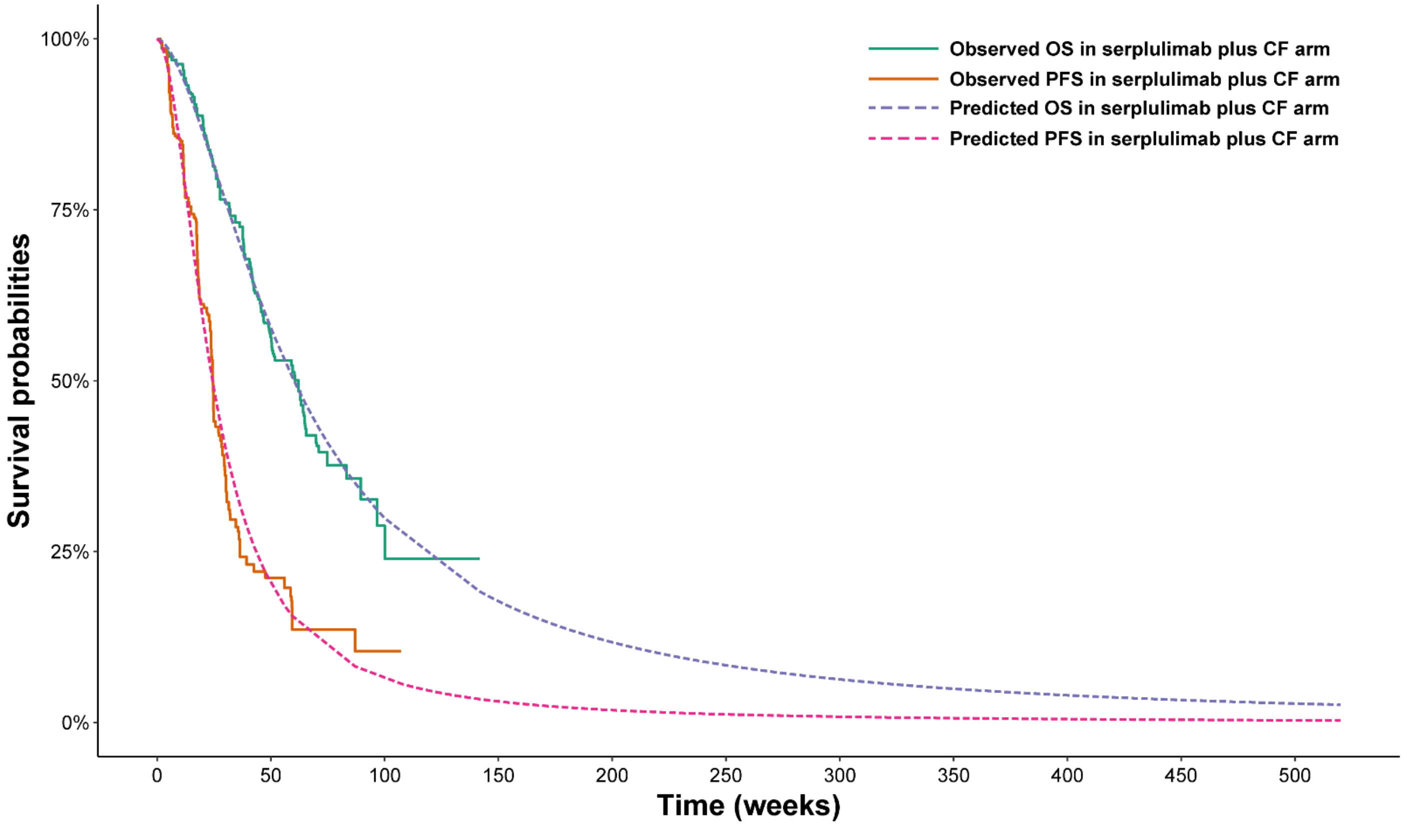

(D) Model-fitted versus original K-M curves for CF, Log-logistic model was used to fit the OS and PFS K-M curves for CF in patients with PD-L1 expression level of 1 ≤ CPS < 10.

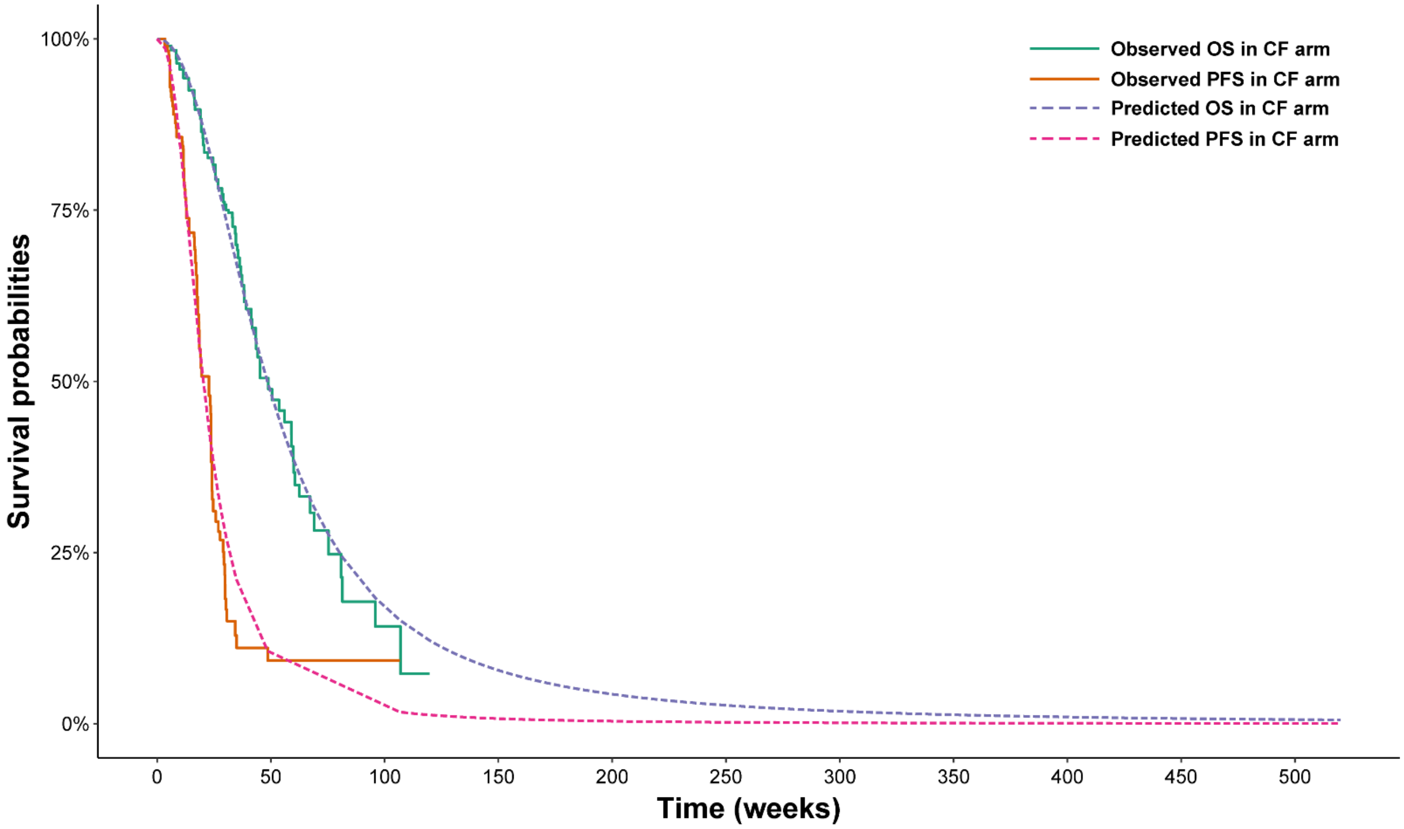

(E) Model-fitted versus original K-M curves for serplulimab plus CF, Lognormal model was used to fit the OS and PFS K-M curves for serplulimab plus CF in patients with PD-L1 CPS ≥ 10.

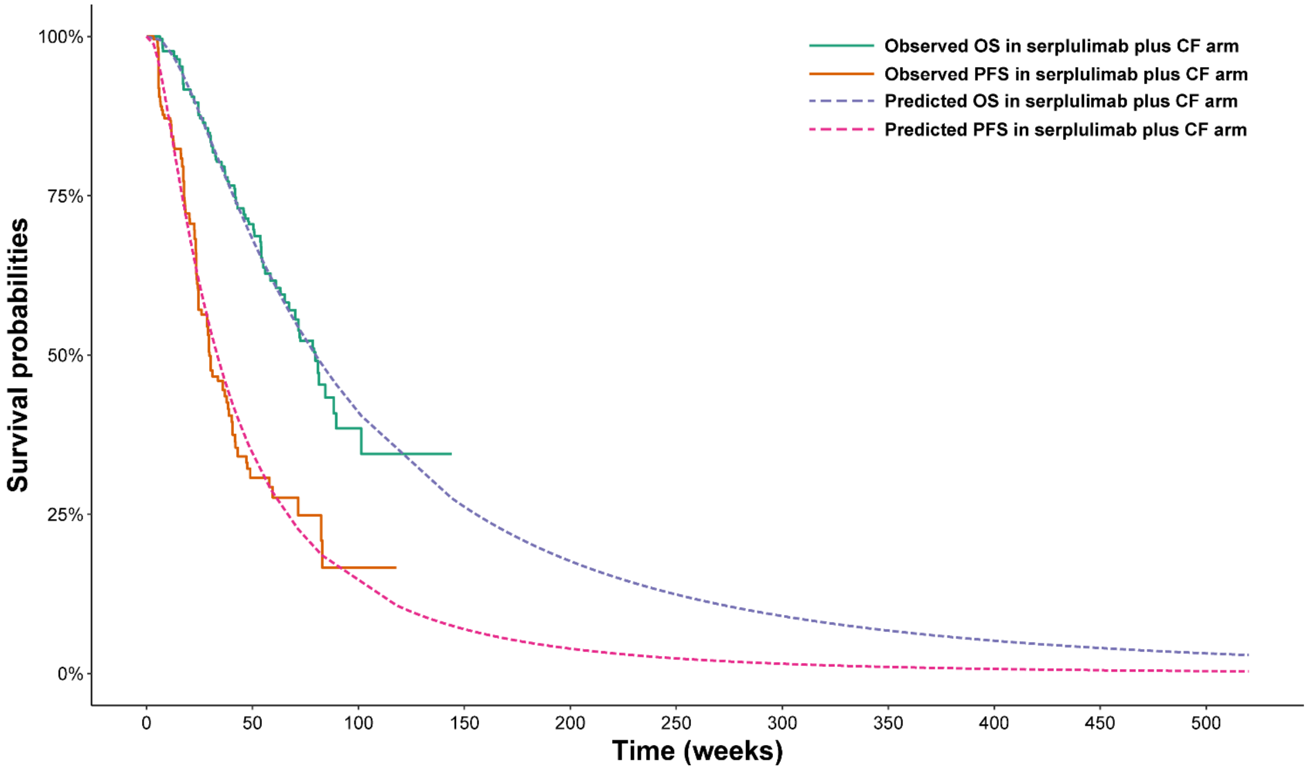

(F) Model-fitted versus original K-M curves for CF, Log-logistic model was used to fit the OS and PFS K-M curves for CF in patients with PD-L1 CPS ≥ 10.

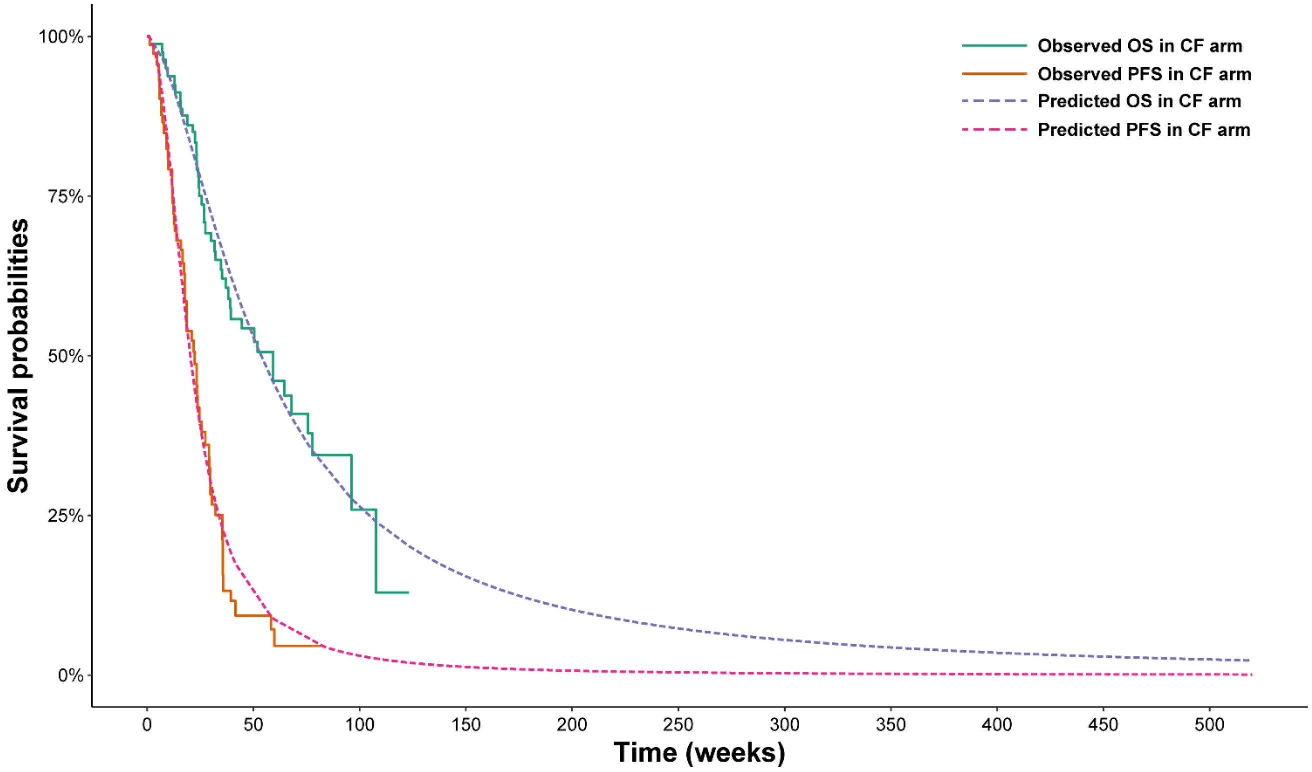

Supplementary Figure 1. Model fitting analysis. To obtain the best model fit, the following investigations were carried out using serplulimab-CF or CF alone as the model fit baseline, respectively. CF, cisplatin plus 5-fluorouracil; CPS, combined positive score.

(A) Overall patients

(B) Patients with PD-L1 expression level of $1 \leq CPS < 10$.

(C) Patients with PD-L1 CPS ≥ 10

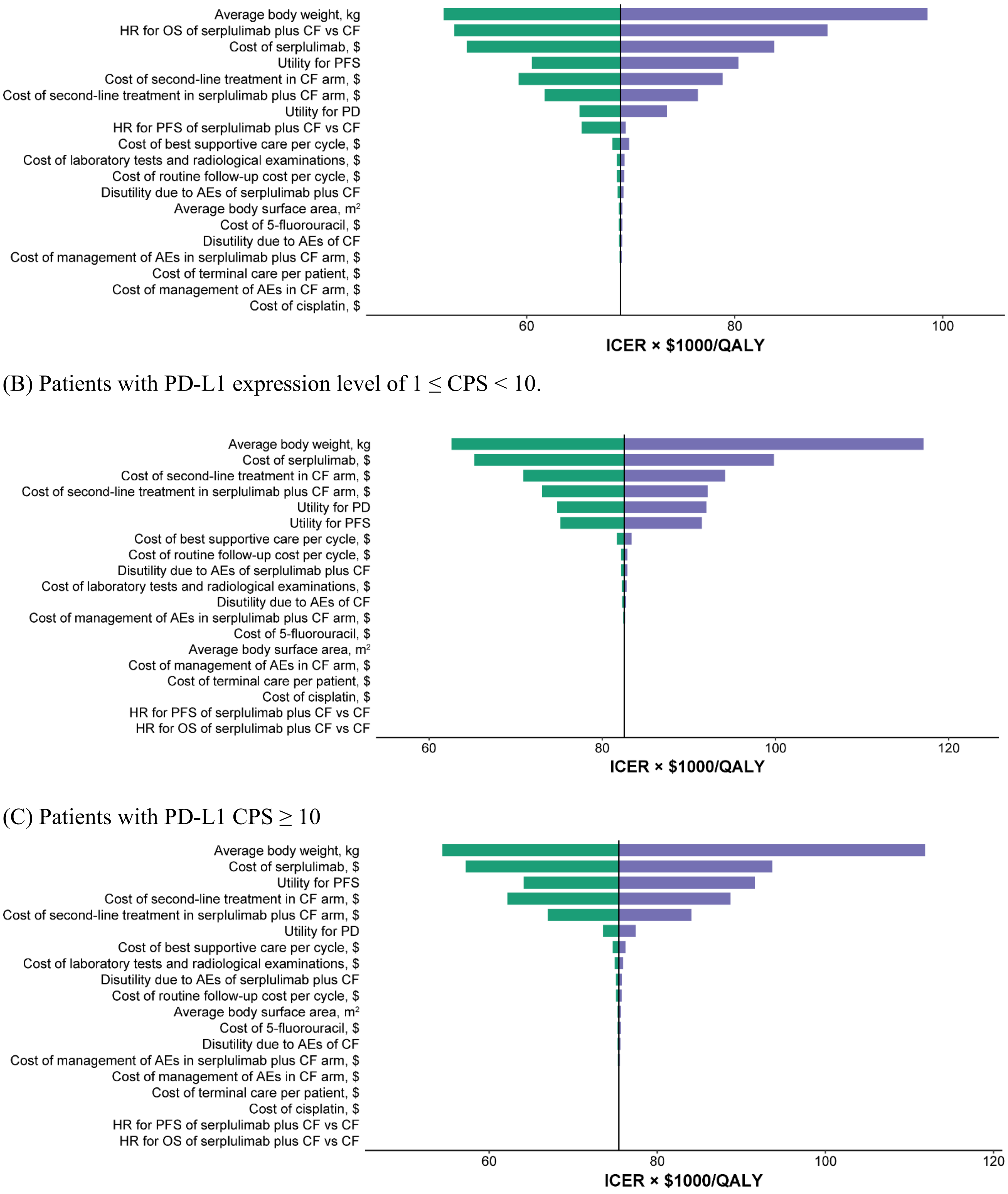

Supplementary Figure 2. Tornado diagram of one-way sensitivity analyses of serplulimab plus CF versus CF. CF, cisplatin plus 5-fluorouracil; ICER, Incremental cost-effectiveness ratio; OS, overall survival; HR, hazard ratio; PD, progressed disease; PFS, progression-free survival; AEs, adverse events.

(A) represents the impacts of varying body weight

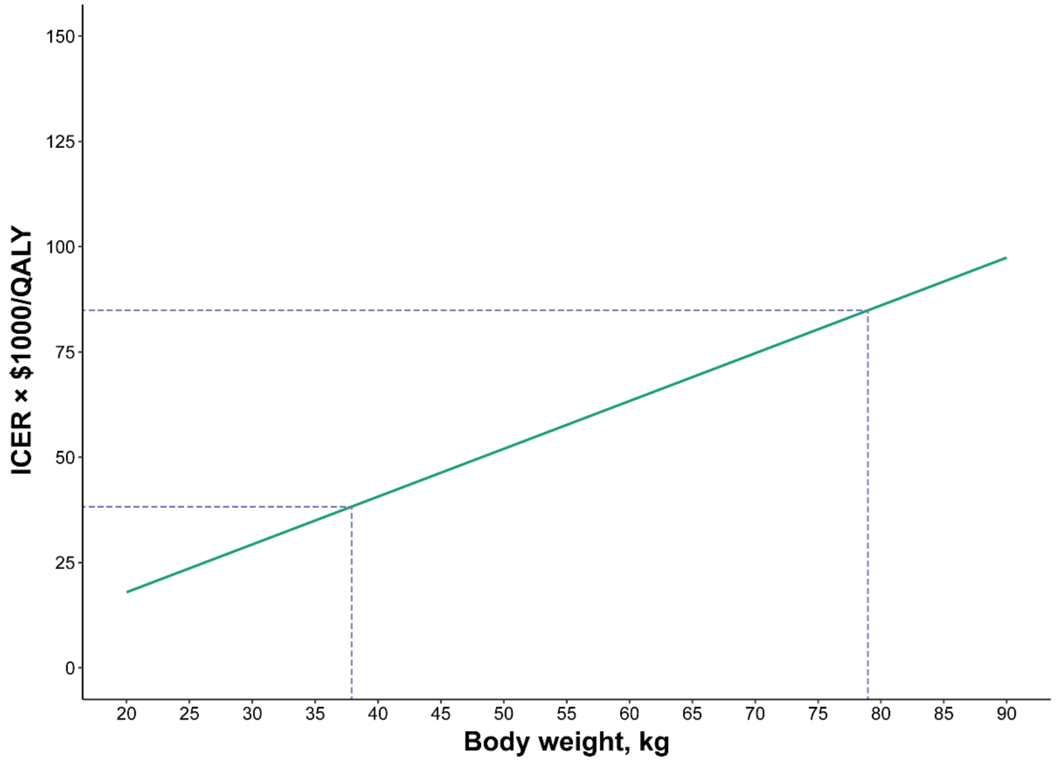

(B) represents the impacts of serplulimab cost

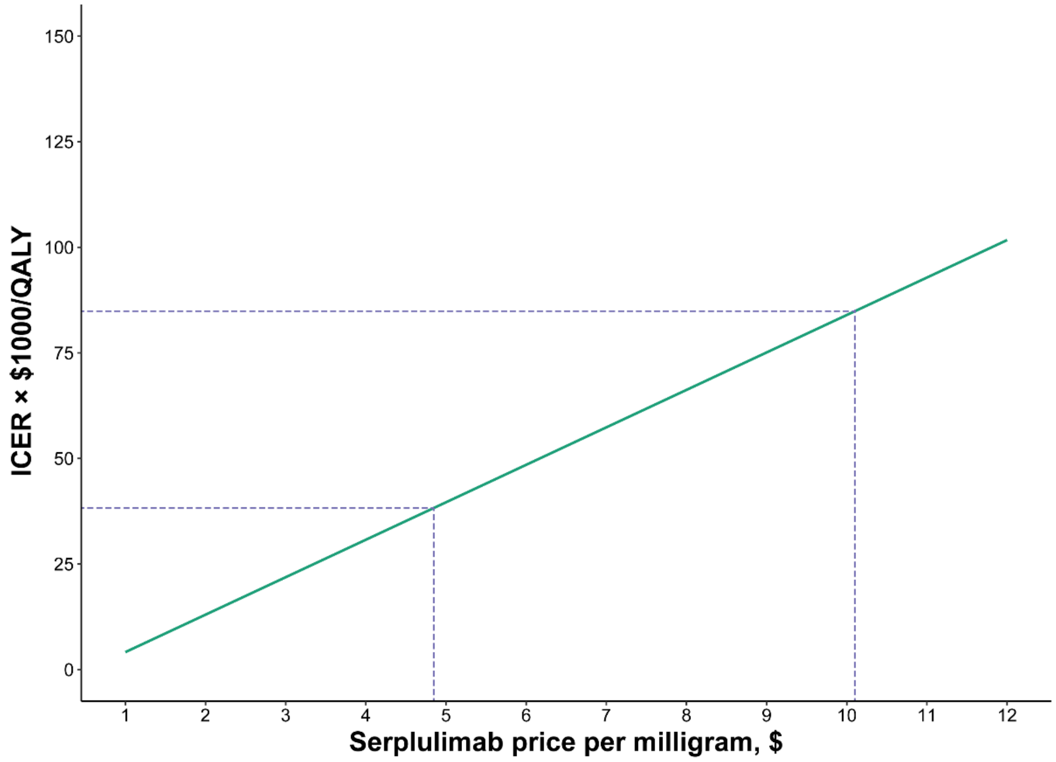

Supplementary Figure 3. Analyses result of ICER when varying body weight and cost of serplulimab in overall PD-L1-positive ESCC patients. Graphs represent the ICERs of serplulimab plus CF.

Supplementary Table 1. Estimated parameters and AIC and BIC values from each survival model.

(A) Overall PD-L1-positive ESCC patients

Strategies	Distributions	Parameters	est	se	L95%	U95%	AIC	BIC
**Results of OS**								
**Serplulimab plus CF**	Exponential	rate	0.0094	0.0007	0.0080	0.0109	1884.696	1888.605
	Weibull	shape	1.3941	0.0886	1.2308	1.5789	1863.392	1871.208
		scale	0.0019	0.0007	0.0009	0.0039		
	Gamma	shape	1.6496	0.1519	1.3772	1.9760	1860.279	1868.095
		rate	0.0193	0.0026	0.0147	0.0252		
	Lognormal	meanlog	4.2447	0.0724	4.1027	4.3866	1863.02	1870.836
		sdlog	1.0636	0.0619	0.9490	1.1921		
	Gompertz	shape	0.0081	0.0027	0.0029	0.0134	1877.988	1885.804
		rate	0.0070	0.0009	0.0055	0.0090		
	Log-logistic	shape	1.7290	0.1122	1.5224	1.9635	**1856.273**	**1864.089**
		scale	67.4343	4.2972	59.5166	76.4053		
	Generalized gamma	mu	4.3764	0.0882	4.2036	4.5492	1860.454	1872.179
		sigma	0.8920	0.0947	0.7243	1.0984		
		Q	0.4762	0.2208	0.0434	0.9089		
**CF**	Exponential	rate	0.0134	0.0013	0.0111	0.0162	1128.223	1131.433
	Weibull	shape	1.5044	0.1177	1.2906	1.7536	1107.873	1114.292
		scale	0.0018	0.0009	0.0007	0.0046		
	Gamma	shape	1.8857	0.2261	1.4907	2.3854	1106.08	1112.499
		rate	0.0303	0.0049	0.0221	0.0416		
	Lognormal	meanlog	3.9133	0.0793	3.7578	4.0688	1109.749	1116.168
		sdlog	0.9260	0.0664	0.8046	1.0659		
	Gompertz	shape	0.0135	0.0036	0.0065	0.0206	1117.441	1123.86
		rate	0.0086	0.0014	0.0062	0.0119		
	Log-logistic	shape	1.9429	0.1570	1.6583	2.2763	**1106.197**	**1112.616**
		scale	49.9632	3.6399	43.3151	57.6317		
	Generalized gamma	mu	4.0826	0.1085	3.8700	4.2953	1107.719	1117.348
		sigma	0.7775	0.0943	0.6130	0.9860		
		Q	0.5577	0.2816	0.0058	1.1096		
**Results of PFS**								
**Serplulimab plus CF**	Exponential	rate	0.0232	0.0016	0.0204	0.0265	2135.464	2139.372
	Weibull	shape	1.2409	0.0636	1.1222	1.3720	2121.674	2129.49
		scale	0.0097	0.0024	0.0061	0.0157		
	Gamma	shape	1.5178	0.1223	1.2961	1.7775	2113.148	2120.964
		rate	0.0392	0.0044	0.0316	0.0488		
	Lognormal	meanlog	3.3373	0.0587	3.2222	3.4523	**2091.961**	**2099.778**
		sdlog	0.9854	0.0480	0.8958	1.0841		
	Gompertz	shape	0.0018	0.0032	-0.0045	0.0081	2137.165	2144.981
		rate	0.0223	0.0022	0.0184	0.0271		
	Log-logistic	shape	1.7792	0.0984	1.5965	1.9830	2092.34	2100.157
		scale	27.8635	1.5595	24.9687	31.0939		
	Generalized gamma	mu	3.2958	0.1030	3.0940	3.4976	2093.715	2105.439
		sigma	0.9999	0.0561	0.8958	1.1161		

		Q	-0.1018	0.2059	-0.5053	0.3017		
**CF**	Exponential	rate	0.0353	0.0030	0.0298	0.0417	1183.917	1187.126
	Weibull	shape	1.3963	0.0855	1.2384	1.5743	1161.776	1168.195
		scale	0.0091	0.0028	0.0050	0.0168		
	Gamma	shape	2.0538	0.2235	1.6593	2.5422	1149.174	1155.593
		rate	0.0792	0.0105	0.0611	0.1026		
	Lognormal	meanlog	3.0044	0.0614	2.8840	3.1248	1134.83	1141.249
		sdlog	0.7694	0.0472	0.6822	0.8678		
	Gompertz	shape	0.0051	0.0042	-0.0032	0.0133	1184.559	1190.978
		rate	0.0321	0.0038	0.0255	0.0405		
	Log-logistic	shape	2.3436	0.1672	2.0377	2.6955	**1129.623**	**1136.042**
		scale	20.2701	1.1683	18.1049	22.6942		
	Generalized gamma	mu	2.9837	0.0967	2.7941	3.1733	1136.752	1146.38
		sigma	0.7732	0.0493	0.6823	0.8762		
		Q	-0.0586	0.2110	-0.4722	0.3550		

(B) Patients with PD-L1 expression level of 1 ≤ CPS < 10.

**Strategies**	**Distributions**	**Parameters**	**est**	**se**	**L95%**	**U95%**	**AIC**	**BIC**
**Results of OS**								
**Serplulimab plus CF**	Exponential	rate	0.0107	0.0011	0.0088	0.0130	1098.162	1101.49
	Weibull	shape	1.3448	0.1109	1.1442	1.5807	1088.978	1095.634
		scale	0.0027	0.0012	0.0011	0.0066		
	Gamma	shape	1.5541	0.1853	1.2303	1.9632	1087.788	1094.443
		rate	0.0200	0.0036	0.0141	0.0283		
	Lognormal	meanlog	4.1321	0.0982	3.9396	4.3247	1092.869	1099.525
		sdlog	1.1155	0.0838	0.9627	1.2925		
	Gompertz	shape	0.0076	0.0036	0.0005	0.0147	1095.968	1102.624
		rate	0.0083	0.0014	0.0060	0.0115		
	Log-logistic	shape	1.6812	0.1423	1.4243	1.9845	**1085.852**	**1092.508**
		scale	60.2209	5.1094	50.9951	71.1159		
	Generalized gamma	mu	4.3123	0.1123	4.0921	4.5325	1089.441	1099.424
		sigma	0.8654	0.1207	0.6585	1.1375		
		Q	0.6397	0.2740	0.1026	1.1768		
**CF**	Exponential	rate	0.0140	0.0018	0.0109	0.0180	644.781	647.425
	Weibull	shape	1.6629	0.1670	1.3658	2.0248	626.925	632.214
		scale	0.0010	0.0007	0.0003	0.0039		
	Gamma	shape	2.2225	0.3534	1.6273	3.0353	626.396	631.685
		rate	0.0382	0.0078	0.0256	0.0571		
	Lognormal	meanlog	3.8747	0.0940	3.6906	4.0589	628.699	633.988
		sdlog	0.8277	0.0772	0.6894	0.9938		
	Gompertz	shape	0.0175	0.0047	0.0083	0.0266	634.5	639.789
		rate	0.0079	0.0017	0.0051	0.0122		
	Log-logistic	shape	2.1860	0.2320	1.7755	2.6914	**625.902**	**631.191**
		scale	48.4765	4.1694	40.9562	57.3776		
	Generalized gamma	mu	4.0488	0.1286	3.7967	4.3010	627.879	635.812
		sigma	0.6847	0.1061	0.5053	0.9277		
		Q	0.6163	0.3618	-0.0929	1.3255		

**Results of PFS**								
**Serplulimab plus CF**	Exponential	rate	0.0272	0.0024	0.0230	0.0323	1207.962	1211.29
	Weibull	shape	1.2888	0.0847	1.1331	1.4660	1197.002	1203.657
		scale	0.0099	0.0031	0.0053	0.0184		
	Gamma	shape	1.6256	0.1725	1.3203	2.0014	1191.275	1197.931
		rate	0.0493	0.0070	0.0373	0.0650		
	Lognormal	meanlog	3.1947	0.0739	3.0498	3.3396	1182.6	1189.256
		sdlog	0.9396	0.0592	0.8304	1.0632		
	Gompertz	shape	0.0040	0.0044	-0.0045	0.0126	1209.151	1215.806
		rate	0.0251	0.0032	0.0196	0.0322		
	Log-logistic	shape	1.8984	0.1377	1.6469	2.1884	**1180.04**	**1186.695**
		scale	24.3930	1.6919	21.2925	27.9451		
	Generalized gamma	mu	3.2322	0.1196	2.9978	3.4666	1184.445	1194.429
		sigma	0.9248	0.0698	0.7976	1.0722		
		Q	0.0962	0.2424	-0.3789	0.5713		
**CF**	Exponential	rate	0.0335	0.0039	0.0266	0.0421	643.989	646.633
	Weibull	shape	1.3429	0.1098	1.1441	1.5763	635.288	640.577
		scale	0.0104	0.0042	0.0047	0.0229		
	Gamma	shape	2.0325	0.3000	1.5219	2.7144	626.494	631.782
		rate	0.0760	0.0139	0.0531	0.1086		
	Lognormal	meanlog	3.0305	0.0807	2.8722	3.1888	612.05	617.338
		sdlog	0.7517	0.0633	0.6372	0.8867		
	Gompertz	shape	-0.0002	0.0057	-0.0114	0.0110	645.988	651.276
		rate	0.0336	0.0053	0.0247	0.0457		
	Log-logistic	shape	2.4457	0.2408	2.0165	2.9662	**607.524**	**612.813**
		scale	20.3446	1.4937	17.6179	23.4932		
	Generalized gamma	mu	2.8893	0.1291	2.6364	3.1422	611.998	619.931
		sigma	0.7586	0.0639	0.6432	0.8948		
		Q	-0.4102	0.2936	-0.9856	0.1652		

(C) Patients with PD-L1 CPS ≥ 10

**Strategies**	**Distributions**	**Parameters**	**est**	**se**	**L95%**	**U95%**	**AIC**	**BIC**
**Results of OS**								
**Serplulimab plus CF**	Exponential	rate	0.0078	0.0010	0.0062	0.0100	785.839	788.926
	Weibull	shape	1.4647	0.1462	1.2045	1.7811	775.633	781.808
		scale	0.0011	0.0007	0.0003	0.0039		
	Gamma	shape	1.7899	0.2594	1.3473	2.3778	773.635	779.81
		rate	0.0185	0.0040	0.0121	0.0282		
	Lognormal	meanlog	4.3799	0.1064	4.1713	4.5885	**770.683**	**776.858**
		sdlog	0.9887	0.0907	0.8260	1.1834		
	Gompertz	shape	0.0088	0.0040	0.0010	0.0166	783.25	789.426
		rate	0.0056	0.0012	0.0037	0.0084		
	Log-logistic	shape	1.7951	0.1818	1.4719	2.1893	771.888	778.063
		scale	78.0037	7.5484	64.5275	94.2943		
	Generalized gamma	mu	4.3697	0.1817	4.0135	4.7259	772.678	781.941
		sigma	0.9977	0.1567	0.7333	1.3574		
		Q	-0.0338	0.4821	-0.9786	0.9111		

**CF**	Exponential	rate	0.0123	0.0019	0.0092	0.0165	476.925	479.294
	Weibull	shape	1.3111	0.1639	1.0262	1.6750	474.786	479.525
		scale	0.0035	0.0024	0.0009	0.0134		
	Gamma	shape	1.5121	0.2764	1.0569	2.1636	474.27	479.009
		rate	0.0214	0.0056	0.0128	0.0359		
	Lognormal	meanlog	3.9976	0.1450	3.7134	4.2817	475.844	480.583
		sdlog	1.0961	0.1237	0.8786	1.3675		
	Gompertz	shape	0.0083	0.0057	-0.0028	0.0193	476.905	481.644
		rate	0.0093	0.0024	0.0057	0.0154		
	Log-logistic	shape	1.6515	0.2079	1.2903	2.1138	**473.956**	**478.695**
		scale	53.6336	7.0551	41.4446	69.4075		
	Generalized gamma	mu	4.1840	0.1862	3.8190	4.5490	475.974	483.082
		sigma	0.9005	0.1784	0.6107	1.3279		
		Q	0.5693	0.4322	-0.2777	1.4164		
**Results of PFS**								
**Serplulimab plus CF**	Exponential	rate	0.0194	0.0020	0.0159	0.0237	940.851	943.939
	Weibull	shape	1.2208	0.0980	1.0431	1.4289	937.274	943.449
		scale	0.0085	0.0033	0.0040	0.0181		
	Gamma	shape	1.4621	0.1801	1.1485	1.8613	934.146	940.321
		rate	0.0317	0.0055	0.0225	0.0445		
	Lognormal	meanlog	3.5080	0.0924	3.3269	3.6890	**923.759**	**929.934**
		sdlog	1.0168	0.0766	0.8771	1.1786		
	Gompertz	shape	0.0013	0.0047	-0.0078	0.0104	942.777	948.952
		rate	0.0188	0.0029	0.0139	0.0255		
	Log-logistic	shape	1.6948	0.1434	1.4358	2.0006	926.078	932.253
		scale	32.9911	2.9509	27.6860	39.3127		
	Generalized gamma	mu	3.3417	0.1928	2.9637	3.7196	924.698	933.961
		sigma	1.0591	0.0843	0.9062	1.2378		
		Q	-0.3919	0.3868	-1.1500	0.3663		
**CF**	Exponential	rate	0.0362	0.0047	0.0281	0.0466	520.188	522.557
	Weibull	shape	1.4610	0.1415	1.2083	1.7665	509.693	514.431
		scale	0.0076	0.0039	0.0028	0.0206		
	Gamma	shape	1.9813	0.3234	1.4388	2.7282	507.325	512.064
		rate	0.0775	0.0154	0.0525	0.1143		
	Lognormal	meanlog	2.9854	0.1004	2.7887	3.1821	508.744	513.483
		sdlog	0.8294	0.0762	0.6928	0.9930		
	Gompertz	shape	0.0154	0.0072	0.0013	0.0295	518.16	522.899
		rate	0.0274	0.0054	0.0186	0.0402		
	Log-logistic	shape	2.1759	0.2315	1.7664	2.6805	**506.224**	**510.963**
		scale	20.3428	1.9295	16.8919	24.4988		
	Generalized gamma	mu	3.1487	0.1428	2.8687	3.4286	508.571	515.679
		sigma	0.7584	0.0847	0.6093	0.9439		
		Q	0.4506	0.3030	-0.1433	1.0445		

Abbreviations: AIC, Akaike information criterion; BIC, Bayesian Information Criterion; CF, cisplatin plus 5-fluorouracil; OS, overall survival; PFS, progression-free survival.

Supplementary Table 2. Associated costs and disutility of grade ≥ 3 treatment-related adverse events.

Adverse Event[a]	No. of patients (%)[b]	Costs in 2022 USD[c]	Reference	Disutility	Reference
**Serplulimab plus CF**					
Anaemia	67 (18%)	3,961	[1]	0.072	[2]
Decreased neutrophil count	71 (19%)	3,439	[1]	0.348	[3]
Decreased white blood cell count	43 (11%)	5,091	[4]	0.072	[3]
Decreased platelet count	15 (4%)	7,482	[5]	0.108	[6]
**Total**		2,201		0.090	
**CF**					
Anaemia	34 (20%)	3,961	[1]	0.072	[2]
Decreased neutrophil count	29 (17%)	3,439	[1]	0.348	[3]
Decreased white blood cell count	11 (7%)	5,091	[4]	0.072	[3]
Decreased platelet count	3 (2%)	7,482	[5]	0.108	[6]
**Total**		1,862		0.081	

[a]Our analysis only included and evaluated grade ≥ 3 treatment-related adverse events.

[b]Number within treatment arm: serplulimab plus CF (N = 368), CF (N = 183).

[c]Calculated as an average cost of toxicity using the weighted frequency of occurrence. This value was used in the base-case model.

References

[1] Wu B, Dong B, Xu Y, Zhang Q, Shen J, Chen H, et al. Economic Evaluation of First-Line Treatments for Metastatic Renal Cell Carcinoma: A Cost-Effectiveness Analysis in a Health Resource-Limited Setting. *PLoS One* (2012) 7(3):e32530. doi: 10.1371/journal.pone.0032530

[2] Freeman K, Connock M, Cummins E, Gurung T, Taylor-Phillips S, Court R, et al. Fluorouracil Plasma Monitoring: Systematic Review and Economic Evaluation of the My5-Fu Assay for Guiding Dose Adjustment in Patients Receiving Fluorouracil Chemotherapy by Continuous Infusion. *Health Technol Assess* (2015) 19(91):1-321, v-vi. doi: 10.3310/hta19910

[3] Nafees B, Lloyd AJ, Dewilde S, Rajan N, Lorenzo M. Health State Utilities in Non-Small Cell Lung Cancer: An International Study. *Asia Pac J Clin Oncol* (2017) 13(5):e195-e203. doi: 10.1111/ajco.12477

[4] Wong W, Yim YM, Kim A, Cloutier M, Gauthier-Loiselle M, Gagnon-Sanschagrin P, et al. Assessment of Costs Associated with Adverse Events in Patients with Cancer. *PLoS One* (2018) 13(4):e0196007. doi: 10.1371/journal.pone.0196007

[5] Zheng H, Xie L, Zhan M, Wen F, Xu T, Li Q. Cost-Effectiveness Analysis of the Addition of Bevacizumab to Chemotherapy as Induction and Maintenance Therapy for Metastatic Non-Squamous Non-Small-Cell Lung Cancer. *Clin Transl Oncol* (2018) 20(3):286-93. doi: 10.1007/s12094-017-1715-1

[6] Konidaris G, Paul E, Kuznik A, Keeping S, Chen CI, Sasane M, et al. Assessing the Value of Cemiplimab for Adults with Advanced Cutaneous Squamous Cell Carcinoma: A Cost-Effectiveness Analysis. *Value Health* (2021) 24(3):377-87. doi: 10.1016/j.jval.2020.09.014
